# An Electrostatic-Barrier-Forming Window that Captures Airborne Pollen Grains to Prevent Pollinosis

**DOI:** 10.3390/ijerph14010082

**Published:** 2017-01-15

**Authors:** Yoshihiro Takikawa, Yoshinori Matsuda, Teruo Nonomura, Koji Kakutani, Shin-Ichi Kusakari, Hideyoshi Toyoda

**Affiliations:** 1 Plant Center, Institute of Advanced Technology, Kindai University, Wakayama 642-0017, Japan; takikawa@waka.kindai.ac.jp; 2 Laboratory of Phytoprotection Science and Technology, Faculty of Agriculture, Kindai University, Nara 631-8505, Japan; nonomura@nara.kindai.ac.jp; 3 Pharmaceutical Research and Technology Institute, Kindai University, Osaka 577-8502, Japan; kakutani@kindai.ac.jp; 4 Research Association of Electric Field Screen Supporters, Nara 631-8505, Japan; kusakari@mbox.kannousuiken-osaka.or.jp (S.-I.K.); toyoda@nara.kindai.ac.jp (H.T.)

**Keywords:** *Cryptomeria japonica*, electric field, pollen capture

## Abstract

An electrostatic-barrier-forming window (EBW) was devised to capture airborne pollen, which can cause allergic pollinosis. The EBW consisted of three layers of insulated conductor wires (ICWs) and two voltage generators that supplied negative charges to the two outer ICW layers and a positive charge to the middle ICW layer. The ICWs generated an attractive force that captured pollen of the Japanese cedar, *Cryptomeria japonica*, from air blown through the EBW. The attractive force was directly proportional to the applied voltage. At ≥3.5 kV, the EBW exerted sufficient force to capture all pollen carried at an air flow of 3 m/s, and pollen-free air passed through the EBW. The findings demonstrated that the electrostatic barrier that formed inside the EBW was very effective at capturing airborne pollen; thus, it could allow a home to remain pollen-free and healthy despite continuous pollen exposure.

## 1. Introduction

Pollinosis is an allergic disease caused by airborne pollen. In Japan, pollen from the Japanese cedar (*Cryptomeria japonica*) and Japanese cypress (*Chamaecyparis obtusa*) are the major pollinosis allergens and are widespread nationwide from February to May. According to government research [1], more than 2.5 million people in Japan are sensitive to these pollen allergens and develop symptoms such as headache, eye congestion and itchiness, repeated sneezing with runny nose, throat irritation and pain, physical weariness, and reduced concentration during the annual pollen season [2]. Normally, people let fresh air into a room by opening windows. To improve the quality of life of pollinosis sufferers during pollen season, we sought to obtain pollen-free space in homes. To this end, we develop an electrostatic-barrier-forming window (EBW) that prevents pollen grains in the air from entering a house using electrostatic attraction. We originally developed similar barriers to trap insect pests for safe crop production and preservation [3,4,5]. In the present study, this concept was expanded to a new electrostatic apparatus for capturing airborne pollen grains. The structure of the proposed apparatus is very simple and easy to construct, and most importantly it can operate continuously for a long period of time with low energy consumption.

## 2. Materials and Methods

**Figure 1:**
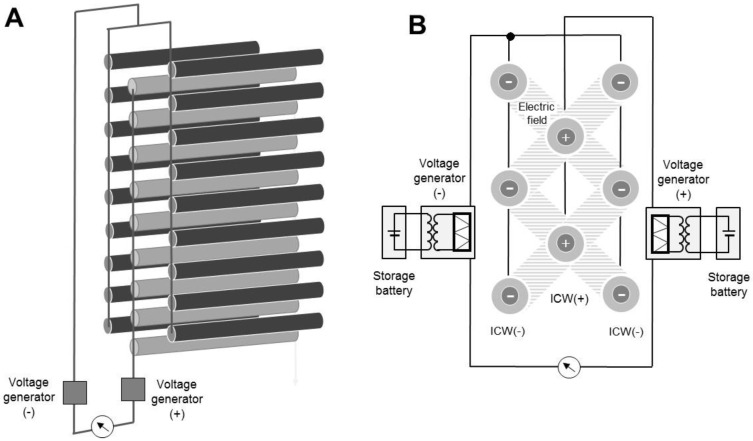
Structure (**A**) and cross-sectional view (**B**) of the three insulated conductor wire (ICW) layers of the electrostatic-barrier-forming window (EBW).

The EBW uses insulated conductor wires (ICWs) as electrodes to form electric fields. Iron wires (2 mm diameter, 15–55 cm length) were insulated by passing them through a transparent insulator vinyl sleeve (1 mm thickness; bulk resistivity 1 × 10^9^ Ω·cm) and were used to construct the EBW. The EBW consisted of three layers of ICWs in parallel arrays and two electrostatic direct current (DC) voltage generators (DMS-P and DMS-N; Max Electronics, Tokyo, Japan) that supplied negative and positive voltages to the ICWs (Figure 1A). Within each layer, the ICWs were parallel at 5 mm intervals and connected to each other and to the negative or positive voltage generator. The negatively and positively charged ICWs are represented as ICW(−) and ICW(+), respectively. The EBW consisted a central ICW(+) layer with an ICW(−) layer on either side. The layers were parallel and 2 mm apart, and the ICWs in the different layers were offset from each other. The generators were linked to each other to make an electric circuit producing electric fields between the ICW(−) and ICW(+) (Figure 1B). Both generators were operated with 12 V storage batteries, with power supplied by a 15 W solar panel, to supply equal negative and positive voltages to the ICWs. In this system, free electrons from ICW(+) were pushed out to ICW(−), and the opposite surface charges on the ICWs acted as dipoles that formed an electric field between them [6]. A galvanometer (PC7000; Sanwa Electric Instrument, Tokyo, Japan) was integrated into the line between the voltage generators.

**Figure 2:**
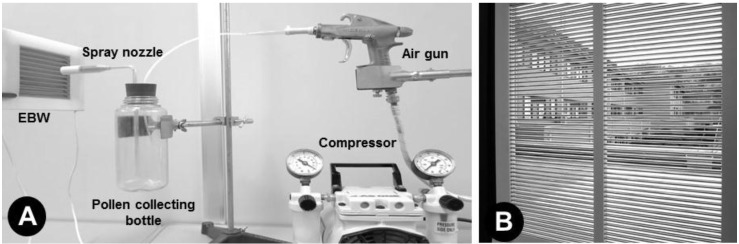
Test-size EBW used for the pollen-blowing test (**A**) and window-size EBW installed on a laboratory window (**B**).

Japanese cedar (*C. japonica*) pollen was purchased from Wako Pure Chemical Industries (Osaka, Japan) and used as test pollen. Fresh pollen grains were placed in a collection bottle linked to an air compressor (Figure 2A). The ICWs of the test-size EBW (20 × 20 cm) were charged with the same negative and positive voltages (0.5–4.0 kV) to determine the voltage range for capturing all of the test pollen. Pollen grains in the bottle were blown into the space between the ICWs by sending compressed air (1.5 kg/cm^2^) through the tip of the spray nozzle. The distance between the tip of the nozzle and the surface of the ICW was altered to create different wind speeds (1–3 m/s). Wind speed was measured at the surface of the ICW using a sensitive anemometer (Climomaster 6533; Kanomax, Tokyo, Japan). A surface-adhesive plate (20 × 20 cm) (Insect-Trapping Adhesive Plate ESC0641; Earth Environmental Service, Tokyo, Japan) was placed on the other side of the EBW to collect pollen passing through the EBW. After the pollen was blown toward the EBW, we counted the number of pollen grains on all of the ICWs (for successfully trapped pollen) and the adhesive plate (for non-trapped pollen) to determine the rate of capture at each voltage and wind speed. Experiments were repeated five times, and data are presented as mean ± SD. Significant differences among the data were analyzed using Tukey’s method (see Table 1). A sample process of pollen attraction was also recorded using a digital EOS camera (Canon, Tokyo, Japan) equipped with a high-fidelity digital microscope (KH-2700; Hirox, Tokyo, Japan) [7].

For practical testing, a window-size EBW (60 × 60 cm) (Figure 2B) was installed on each of two opposite windows in our laboratory. An adjacent room of the same size and structure was used as a control with no EBW. Three pollen detectors (Pollen Sensor PS2; Shinyei Technology, Kobe, Japan) were placed in each room to monitor the pollen entering through the windows. Monitoring continued for 1 month, and the same complete experiment was repeated three times in the period of the most active *C. japonica* pollen disperse in the year (February to April in 2016).

## 3. Results and Discussion

We constructed an EBW with multiple gap-free electric fields because successful pollen capture depends on the formation of an electrostatic barrier with no spaces through which pollen can pass. An essential step in forming the electric fields was charging the insulated electrodes. High voltages produced through the Cockcroft circuit [8] of two voltage generators were used to electrify both electrodes by adding electrons to ICW(−) and pushing electrons out of ICW(+).

Table 1 lists the percentage of the pollen trapped by ICWs charged at different voltages and the adhesive plate placed over the EBW. The number of plate-trapped pollen grains decreased with the applied voltage. In the electric field, pollen was subject to the electrostatic attractive force and air flow force, and their direction was determined by the combined vector of these two forces. The ICWs could trap all of the pollen in the electric field generated when charging the electrodes with more than 3.5 kV, while pollen passed through the EBW when the electrodes were charged with lower voltages. Under these conditions, the field force was weaker than the air flow force. These findings indicate that the reduction in the trapping force increased as the voltage decreased. To demonstrate this visually, supplemental video material shows that the EBW can capture pollen blown toward the screen (Supplementary Video S1) . In the video, the airborne pollen looks like a fine mist and the electrostatic barrier completely prevents this mist from passing through the EBW with a sufficient charge.

With regard to the mechanism of the pollen attraction force, in some of our previous research, we developed an electric field between ICWs and a grounded net by placing a grounded metal net on each side of the ICW layer. In that system, ICW(−) pushed free electrons from the surface cuticle layers (conductor) of insects released near ICW(–) to give insects a net positive charge [3,5], whereas insects near ICW(+) gained a net negative charge with the addition of free electrons to the cuticle layer [4]; ultimately, the negatively and positively electrified insects were attracted to the oppositely charged electrodes. In the current study, however, we present an additional explanation of the pollen attraction in the electric field because of the dielectrophoretic movement of the pollen subjected to a non-uniform electric field. Dielectrophoresis is a phenomenon in which a force is exerted on a dielectric particle (oppositely polarized particle) in a non-uniform electric field [9]. This force does not require the particle to be charged, because all particles exhibit dielectrophoretic activity in the presence of a non-uniform electric field. It is clear that the round electrodes used in this study produce a non-uniform electric field so that the pollen becomes polarized dielectrically. According to the dielectrophoresis theory, the polarization of the pollen relative to the surrounding electric field changes along the gradient of the electric field strength. This changeable polarization enables the pollen to move toward the electrodes. In fact, the pollen moved toward the nearest electrode, i.e., in the direction of increasing electric field intensity produced by the electrode. In this study, the electrodes were oppositely charged with equal voltages, so that both electrodes would create the same gradient of field strength. These oppositely charged electrodes exerted the same pollen attraction force. The gradient of the field intensity increased with the voltage applied to the electrodes; eventually, both electrodes created a force strong enough to capture the pollen.

In the second experiment, the EBW was installed on actual room windows to confirm that it effectively prevented airborne pollen from entering a room with good air penetration. Good air flow is vital for efficient ventilation in healthy housing. However, active air circulation also has a downside because the air flow can carry airborne pollen throughout the house. Our primary concern was to eliminate airborne pollen from the air flow passing through the EBW. Capture efficiency was affected by air flow speed. Our findings indicate that the EBW was capable of trapping all of the pollen entering the EBW at an applied voltage of 3.5 kV, even when the pollen was blown by natural wind at 3 m/s (the highest wind speed recorded during the study; data not shown). In fact, the room with the EBWs remained pollen-free throughout the experiment. In comparison, much Japanese cedar and Japanese cypress pollen was found in the unprotected room, an average of 6700.8 ± 3913.9 and 7960.6 ± 1726.1 pollen grains/m^3^ per day, respectively. These pollen levels are sufficient to cause symptoms of pollinosis [2]. Therefore, this study demonstrated that the EBW is a promising pollen precipitator that can completely eliminate pollen from the air flow passing through the apparatus.

In addition to its ability to capture pollen, the low power consumption of our proposed EBW system is important for practical use. Japan is always at risk for frequent, major electric power failures, such as those following earthquakes, which would bring pollen capture to a halt. In the EBW, the voltage generator is the only driving part requiring an electric power supply, and its electric power is 5 W, equivalent to that of a small electric bulb. This enabled the use of a photovoltaic power generation method for supplying the power to the voltage generators. Using this system, we continuously operated two EBWs attached to room windows for a month. During the experiment, the power supply was stable and was not disturbed by changes in weather, so the EBWs constantly performed their pollen-capturing function.

## 4. Conclusions

The primary contribution of this work was the use of basic electrostatics for improving the human environment. The EBW is a unique product developed for this purpose. The structure of the EBW is very simple, and no special technique was required for its construction. The EBW can operate normally for long continuous periods at low electric power consumption. This work demonstrated that the proposed EBW can easily obtain pollen-free living spaces as a precaution to prevent pollinosis.

## Figures and Tables

**Table 1:** Percentage of Japanese cedar pollens captured by the ICWs of the electrostatic barrier forming window (EBW) and adhesive plate placed on the opposite side of the EBW.

Wind Speed (m/s)	Sites of Pollen Trapping	Negative and Positive Voltages (kV) Applied to ICWs						
		0	1	2	2.5	3	3.5	4
0.5	ICWs	4.8 ± 4.0 ^a^	7.8 ± 2.5 ^a^	8.0 ± 4.1 ^a^	59.9 ± 5.6 ^a^	100 ^a^	100	100
	Adhesive plate	95.2 ± 4.0	92.2 ± 2.5	92.0 ± 4.1	40.1 ± 5.6	0	0	0
1	ICWs	6.8 ± 3.9 ^a^	6.2 ± 3.4 ^a^	7.2 ± 2.7 ^a^	22.6 ± 5.3 ^b^	92.9 ± 4.1 ^b^	100	100
	Adhesive plate	93.2 ± 3.9	93.8 ± 3.4	92.8 ± 2.7	77.4 ± 5.3	7.1 ± 4.1	0	0
2	ICWs	4.8 ± 2.4 ^a^	5.3 ± 1.5 ^a^	6.1 ± 3.2 ^a^	20.6 ± 3.3 ^b^	52.3 ± 10.2 ^c^	100	100
	Adhesive plate	95.2 ± 2.4	94.7 ± 1.5	93.9 ± 3.2	79.4 ± 3.3	47.7 ± 10.2	0	0
3	ICWs	5.0 ± 2.3 ^a^	5.5 ± 3.0 ^a^	5.5 ± 3.0 ^a^	17.5 ± 4.3 ^b^	56.1 ± 12.7 ^c^	100	100
	Adhesive plate	95.0 ± 2.3	94.5 ± 3.0	94.5 ± 3.0	82.5 ± 4.3	43.9 ± 12.7	0	0

Pollen grains were blown toward the ICWs, and the number of the pollens on both the ICWs and the adhesive plate were counted to determine the percentage of trapped pollens by ICWs for each voltage and wind speed. The means and standard deviations were calculated from five replicates. The different letters (a–c) on the mean values in each vertical column indicate significant differences (*p* < 0.05) according to Tukey’s method.

## Supplementary Materials

The following are available online at www.mdpi.com/1660-4601/14/1/82/s1, Video S1: Pollen blown toward a non-charged (top) and charged (bottom) EBW.

## References

[1] Ministry of the Environment Government of Japan Website. https://www.env.go.jp/en/headline/53.html/.

[2] Kaneko Y., Motohashi Y., Nakamura H., Endo T., Eboshida A. (2005). Increasing prevalence of Japanese cedar pollinosis: A meta-regression analysis. Int. Arch. Allergy Immunol..

[3] Kakutani K., Matsuda Y., Haneda K., Sekoguchi D., Nonomura T., Kimbara J., Osamura K., Kusakari S., Toyoda H. (2012). An electric field screen prevents captured insects from escaping by depriving bioelectricity generated through insect movements. J. Electrostat..

[4] Matsuda Y., Kakutani K., Nonomura T., Kimbara J., Kusakari S., Osamura K., Toyoda H. (2012). An oppositely charged insect exclusion screen with gap-free multiple electric fields. J. Appl. Physics.

[5] Nonomura T., Matsuda Y., Kakutani K., Kimbara J., Osamura K., Kusakari S., Toyoda H. (2014). Electrostatic measurement of dischargeable electricity and bioelectric potentials produced by muscular movements in flies. J. Electrostat..

[6] Matsuda Y., Ikeda H., Moriura N., Tanaka N., Shimizu K., Oichi W., Nonomura T., Kakutani K., Kusakari S., Higashi K., Toyoda H. (2006). A new spore precipitator with polarized dielectric insulators for physical control of tomato powdery mildew. Phytopathology.

[7] Matsuda Y., Kakutani K.T., Nonomura T., Kimbara J., Osamura K., Kusakari S., Toyoda H. (2015). Safe housing ensured by an electric field screen that excludes insect-net permeating haematophagous mosquitoes carrying human pathogens. J. Physics.

[8] Wegner H.E., Geller E., Moore K., Weil J., Blumel D., Felsenfeld S., Martin T., Rappaport A., Wangner C., Lai B., Taylor R. (2002). Electrical charging generators. McGraw-Hill Encyclopedia of Science and Technology.

[9] Cross J.A., De Barr A.E. (1987). Dielectrophoresis. Electrostatics: Principles, Problems and Applications.

